# Mapping curvature domains in human V4 using CBV-sensitive layer-fMRI at 3T

**DOI:** 10.3389/fnins.2025.1537026

**Published:** 2025-02-26

**Authors:** Elisa Zamboni, Isaac Watson, Rüdiger Stirnberg, Laurentius Huber, Elia Formisano, Rainer Goebel, Aneurin J. Kennerley, Antony B. Morland

**Affiliations:** ^1^School of Psychology, University of Nottingham, Nottingham, United Kingdom; ^2^York Neuroimaging Centre, University of York, York, United Kingdom; ^3^Biomedical Imaging Science Department, Institute of Cardiovascular and Metabolic Medicine, University of Leeds, Leeds, United Kingdom; ^4^German Centre for Neurodegenerative Diseases, Bonn, Germany; ^5^National Institutes of Health, Bethesda, MD, United States; ^6^Department of Cognitive Neuroscience, Maastricht University, Maastricht, Netherlands; ^7^Institute of Sport, Department of Sports and Exercise Sciences, Manchester Metropolitan University, Manchester, United Kingdom; ^8^Department of Psychology, University of York, York, United Kingdom; ^9^York Biomedical Research Institute, University of York, York, United Kingdom

**Keywords:** fMRI, laminar, layers, VASO, visual features, curvature, columns, 3 Tesla

## Abstract

**Introduction:**

A full understanding of how we see our world remains a fundamental research question in vision neuroscience. While topographic profiling has allowed us to identify different visual areas, the exact functional characteristics and organization of areas up in the visual hierarchy (beyond V1 & V2) is still debated. It is hypothesized that visual area V4 represents a vital intermediate stage of processing spatial and curvature information preceding object recognition. Advancements in magnetic resonance imaging hardware and acquisition techniques (e.g., non-BOLD functional MRI) now permits the capture of cortical layer-specific functional properties and organization of the human brain (including the visual system) at high precision.

**Methods:**

Here, we use functional cerebral blood volume measures to study the modularity in how responses to contours (curvature) are organized within area V4 of the human brain. To achieve this at 3 Tesla (a clinically relevant field strength) we utilize optimized high-resolution 3D-Echo Planar Imaging (EPI) Vascular Space Occupancy (VASO) measurements.

**Results:**

Data here provide the first evidence of curvature domains in human V4 that are consistent with previous findings from non-human primates. We show that VASO and BOLD tSNR maps for functional imaging align with high field equivalents, with robust time series of changes to visual stimuli measured across the visual cortex. V4 curvature preference maps for VASO show strong modular organization compared to BOLD imaging contrast. It is noted that BOLD has a much lower sensitivity (due to known venous vasculature weightings) and specificity to stimulus contrast. We show evidence that curvature domains persist across the cortical depth. The work advances our understanding of the role of mid-level area V4 in human processing of curvature and shape features.

**Impact:**

Knowledge of how the functional architecture and hierarchical integration of local contours (curvature) contribute to formation of shapes can inform computational models of object recognition. Techniques described here allow for quantification of individual differences in functional architecture of mid-level visual areas to help drive a better understanding of how changes in functional brain organization relate to difference in visual perception.

## 1 Introduction

By linking both cerebral physiology and functional properties we can better appreciate how our brains interpret the world; specifically, this work aims to develop our understanding concerning “*exactly how does our visual brain recognize objects?*”

The visual cortex is known to have multiple functional areas that can be identified by their visual topography (or retinotopic maps; Inouye, 1909; Holmes, 1918), but also by their functional characteristics (Van Essen, 2003).

Primary visual cortex, V1, has a visual map spanning the contralateral hemifield (Wandell et al., 2009). At the same time, it also shows a modular organization capturing structure in response properties to specific visual attributes. Indeed, both human and non-human primates V1 have ocular dominance columns (Hubel and Wiesel, 1962) and orientation pinwheels (Bonhoeffer and Grinvald, 1991), highlighting a multi-layered and spatially separate organization of different functional properties. Similarly, the second visual area, V2, contains the representation of a quarter of the contralateral hemifield, with the vertical meridian representation matching that of V1, and the horizontal meridian being adjacent to that of area V3 (Hubel and Wiesel, 1965). Functionally, area V2 shows modular organization with spatially grouped neuron’s responses to orientation, color, and depth (DeYoe and Van Essen, 1985; Shipp and Zeki, 1985; Hubel and Livingstone, 1987). The modular properties of V1 and V2 as exhibited in columnar and striped organization have also been shown with mesoscopic MRI at ultra-high field (Tootell and Nasr, 2017; Nasr et al., 2016).

As one moves higher up the visual hierarchy beyond areas V1 and V2, the retinotopic and functional mapping becomes challenging (across both humans and non-human primates). Area V4 tends to show a contralateral hemifield representation (Winawer and Witthoft, 2015), while in macaques the upper and lower quadrants of the visual field are distinctly split into anatomically separate representations (Zeki, 1971). Conversely, human and macaque V4 shows similar functional properties such as processing chromatic information (Tanigawa et al., 2010; Ghose and Ts’o, 2017) and playing a role in transitioning from retinotopic to shape processing (Lawrence et al., 2023; Vernon et al., 2016). It is hypothesized that visual area V4 therefore represents a vital intermediate stage of processing spatial and curvature information (Kobatake and Tanaka, 1994; Pasupathy and Connor, 1999) preceding object recognition.

Most recently, electrophysiological recordings and intrinsic optical imaging studies (in preclinical models) have identified a modular organization of curvature responses in area V4 (Hu et al., 2020; Tang et al., 2020). By implementing a large stimulus set of simple contour shapes varying in their degree of curvature, Tang and colleagues identified alternating regions of curvature preference versus straight lines in anesthetized macaques. These observed curvature domains overlap with regions of V4 showing preference for orientation (Hu et al., 2020). These identified regions are distinct from color domains, suggesting that the interplay of different functional maps could be a key component of shape information processing architecture in the visual brain.

The size of the curvature domains observed by Hu et al. (2020) and Tang et al. (2020) were on the order of 500 μm in diameter, consistent with other functional domain sizes reported within area V4 (Tanigawa et al., 2010). At a more macroscopic level, Yue et al. (2014) have shown the involvement of a widespread network of visual areas underlying curvature processing in the rhesus monkeys using functional magnetic resonance imaging (fMRI) techniques. Using naturalistic and computer-generated stimuli that varied in their curvature content, the authors identified a posterior curvature patch in dorsal V4, a middle curvature patch in the superior temporal sulcus, and an anterior curvature patch in the inferior temporal area, again highlighting the role of area V4 in processing curved stimuli. Yue et al. (2020) replicated these findings using fMRI at high field strength (7 Tesla) by identifying a network of curvature preferring cortical patches in humans which included area V4 alongside areas V3, lateral occipitotemporal cortex, and the fusiform gyrus. While this work informed on the role of human area V4 in processing curvature information, it lacked the granularity and spatial resolution to determine its modular organization of functional response properties.

Advancements in functional magnetic resonance imaging (fMRI) acquisition techniques and hardware now permits the capture of cortical layer-specific functional properties and organization of the human brain (Huber et al., 2023; Haenelt et al., 2023a; Lawrence et al., 2019; Ciris et al., 2014; Uğurbil, 2021; Koopmans et al., 2010; Fracasso et al., 2018; Scheffler and Ehses, 2016). Activation patterns across distinct layers of the cortex represent a fingerprint of feedforward and feedback information processing (Felleman and Van Essen, 1991), thus layer (laminar) fMRI opens the possibility to investigate hierarchical (modular) information flow in the human brain. However, such studies often rely on the use of ultra-high field strength scanners (>7 Tesla), limiting access to specialist centers worldwide. Furthermore, there is need to step away from traditional fMRI sequence contrasts, for example gradient-echo based Blood Oxygenation Level Dependent (GE-BOLD), which are often biased to the surface vasculature (Duvernoy et al., 1981; Olman et al., 2007). Signal spreading across cortical layers and columns (Kennerley et al., 2005; Turner, 2002) is a critical limitation when investigating the mesoscopic characteristics of functional brain properties. This calls for alternative, non-BOLD quantitative contrast mechanisms capable of mitigating signal biases and improve signal interpretability.

Here, we propose the application of an optimized high-resolution 3D-Echo Planar Imaging (EPI) Vascular Space Occupancy (VASO) sequence (Huber et al., 2019; Stirnberg and Stöcker, 2021), a cerebral blood volume-based approach that offers higher layer-based specificity due to its superior sensitivity to the target microvasculature. Taking advantage of the difference in longitudinal relaxation times (T_1_) between brain tissue and blood (Lu et al., 2003; Lu et al., 2013), the contribution of blood is selectively nulled during signal excitation by applying a magnetisation inversion pulse, yet tissue signal is maintained. Thus, during neuronal activity we observe a decrease in MR signal intensity which is proportional to the increase in the volume fraction of blood (or cerebral blood volume, CBV) within an imaged voxel (Attwell and Iadecola, 2002; Lu and van Zijl, 2005, 2012). Importantly, we deploy this method at clinically relevant field strengths (3 Tesla) to probe the fine spatial organization of the human visual system with high precision.

Specifically, we tested whether human V4 shows a modularity in how responses to contours (curvature) are organized consistent with that observed in macaque’s V4. To test this, we used radial frequency (RF) pattern stimuli, closed contour shapes generated by modulating the radius of a circle, that varied in the amount of curvature information expressed (i.e., concentric circles, RF0, for curvature condition, or concentric squares, RF4, for straight line contours). We measured changes in CBV and BOLD contrast in response to these two stimulus types and hypothesized: (1) curvature domains should show distinct response preferences for curved stimuli over straight contours; (2) curvature domains should be organized in curvature domains, with alternating domains showing preference for curved and straight contours; (3) VASO-based curvature domains should yield higher spatial specificity mapping when compared to BOLD-based curvature maps.

Our results from the human brain are consistent with the preclinical measures of functional organization of curvature responses in V4. Thus, this study delivers emerging evidence that functional brain mapping (based on cerebral blood volume contrast) revealing mesoscopic functional organization of the human brain is possible on a broader range of MR systems than previously believed. Furthermore, understanding how the functional architecture and hierarchical integration of local contours (curvature) contribute to formation of shapes can inform computational models of object recognition. Finally, quantifying individual differences in functional architecture of mid-level visual areas can help understanding how changes in functional brain organization relate to differences in visual perception.

## 2 Materials and methods

### 2.1 Participants

Five healthy participants (5 female, 24–45 years old) with normal or corrected-to-normal visual acuity and no history of neurological impairments were recruited for this study. All participants gave written informed consent prior to taking part in the procedure. The study was approved by the York Neuroimaging Centre (YNiC) Research Ethics Committee at the University of York (UK) and in accordance with the Declaration of Helsinki. Each participant underwent a minimum of one (2 h) and a maximum of two (4 h in total, performed 2 weeks apart as test-retest reliability) scanning sessions. To ensure participants comfort throughout the session, we provided pillows, and foam inserts around their head and ears. A further cushion was positioned under their knees to remove any pressure on their back from a relatively prolonged stable supine position. As per standard ethical and safety procedures, participants were provided a buzzer which could be pressed at any time should they need a break or felt they wanted to withdraw from the study.

### 2.2 Imaging parameters

All imaging data were acquired on a Siemens MAGNETOM 3T Prisma scanner at the York Neuroimaging Centre (YNiC), University of York, using a 32-channel 1H head coil for both functional and structural data.

Functional scans were performed using a 3D-EPI based VASO sequence optimized for use at clinical field strengths (Stirnberg and Stöcker, 2021) with a nominal resolution of 1.2 mm isotropic (20 slices, TI1/2/TR/TE = 1145/2115/2610/17.2 ms; pF = 6/8 with POCS#8; flip angle = 30° FLASH GRAPPA 3; bandwidth = 1008 Hz/Px; FoV = 190 mm). Slice position and orientation were adjusted individually to cover the ventrolateral visual cortex.

Retinotopic mapping data was acquired using a standard GE-BOLD EPI sequence (52 slices, 2.5 mm isotropic, TR/TE = 1000/30 ms; pF = 7/8; flip angle = 75° Multi-Band factor = 4; bandwidth = 1786 Hz/Px; FoV = 200 mm).

Whole brain T1-weighted structural images (0.8 mm isotropic) were acquired using standard MP2RAGE (Marques et al., 2010).

### 2.3 Stimuli

Stimuli were presented on a gray background and consisted of concentric radial frequency patterns generated using custom-based Matlab scripts. Specifically, radial frequency patterns (Wilkinson et al., 1998) are defined as:


$$r\,\left(\theta \right)=r_{0}\,\left(1+A\,\left(\sin\omega \,\theta +\Phi \right)\right)$$

The angle around a circle’s perimeter (θ) determines the deviation from a circle by altering frequency (ω) and amplitude (A), while Φ governs rotation of the pattern. Here, we used a radial frequency of zero to generate concentric circles (RF0; curved stimulus), and radial frequency of four to generate rounded squares (RF4; straight stimulus). The latter shows straight contours crossing the horizontal and vertical meridians of the visual field, while the rounded corners still provide a degree of curvature information. The radius (r_0_), determining the size of the stimulus, was set to a maximum of 9 degrees of visual angle. For each radial frequency pattern (RF0 and RF4), two stimuli with reversed contrast were generated, such that during stimulus presentation the two contrasts alternated over time to minimize visual adaptation (Larsson et al., 2016). The experiment was controlled using MATLAB and PsychToolbox 3.0 (Brainard, 1997; Pelli, 1997) and stimuli were presented using a projector and a mirror setup (1920 × 1080 pixels resolution, 120 Hz frame rate) at a viewing distance of 62 cm.

### 2.4 Experimental procedure

The study consisted of one, 2-h session, with one participant performing two, 2-h sessions. Each participant underwent 4 experimental runs (curvature mapping), each lasting approximately 17 min; and 4 retinotopic mapping scans (each lasting 2 min 8 s). The remainder of the session was used to acquire high-resolution structural images and ensure the functional acquisition was capturing the ventrolateral visual cortex.

**FIGURE 1:**
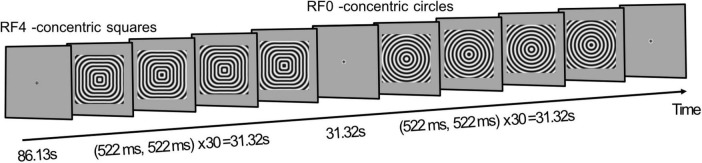
Experimental design. A functional run started with 86.13 s fixation (33× TRs), followed by a 31.32 s block where contrast reversing concentric squares (RF4) are presented. After 31.32 s fixation, another stimulus block, presenting concentric circles (RF0), is shown for 31.32 s. A total of 14 stimulus blocks are displayed per functional run. Participants are required to maintain fixation throughout.

The curvature mapping functional scans used a block-design: following an initial 86.13 s (33× TRs) of fixation, fourteen 31.32 s on-off blocks were presented in an ArBr fashion. Here, during odd blocks concentric radial frequency patterns (Wilkinson et al., 1998) with a radial frequency of zero (RF0; concentric circles) were presented, while even blocks showed RF4 (concentric squares) stimuli. During each on-block, the radial frequency pattern was contrast reversed every 522 ms (Figure 1). Throughout the session participants were required to maintain fixation at the center of the screen.

Retinotopic mapping followed previously described procedures (Welbourne et al., 2018). Briefly, a bar stimulus (width 0.5 degrees) moved in one of eight possible directions (top-bottom, left-right, top left-bottom right, top right-bottom left) within a 10 degrees radius circular aperture. A movement across the full field lasted 16 s, followed by a movement across half the direction for 8 s, and interleaved with a mean luminance blank period for 8 s before starting the cycle again. Participants underwent 4 scans while performing an attentional task, indicating via key press a change in color in the fixation cross.

### 2.5 Structural data analysis

T1-weighted structural data was used for coregistration and 3D cortex reconstruction. Initial gray and white matter segmentation for each hemisphere was obtained using Freesurfer (v6.0)^1^ and manually improved around visual cortex using ITK-Snap (Yushkevich et al., 2006). In order to minimize interpolation resulting from registration that would impact depth-dependent analysis (Huber et al., 2017; Guidi et al., 2020), the structural T1-weighted image of each participant was aligned to the functional, high-resolution VASO data, following https://layerfmri.com/2019/02/11/high-quality-registration/. Computing the inverse of the signal variability in the VASO time series resulted in a derived T1-weighted image (T1w-EPI) with good gray matter-white matter contrast and the same 3D slab dimensions as the functional data. In FSL viewer (Jenkinson et al., 2012) we first manually aligned the T1-weighted whole brain structural to the T1w-EPI slab using Nudge. The resulting transformation matrix was saved and used as input to FSL flirt registration algorithm. Here the T1-weighted structural images were resampled to the T1w-EPI space using spline-interpolation.

To aid investigation of the modular organization of curvature responses in V4, it is essential that tissue segmentation of this regions is accurate. This was achieved by first upsampling (with cubic interpolation; AFNI v.23.0.04, -3dresample command; Cox and Hyde, 1997) the T1-weighted structural images with an upscaling factor of 6 (nominal resolution of 0.2 mm isotropic). This smoothed the curvature and thickness properties of the cortex. We then localized the segmentation process around a “scoop” in both hemispheres: in ITK-Snap we centered a spherical mask around area V4 (derived from retinotopic mapping – see below) and obtain a first definition of cerebrospinal fluid/gray matter (CSF/GM) and gray matter/white matter (GM/WM) tissue boundaries. Manual inspection of the segmentation for each hemisphere was performed to ensure no voxels were mislabelled in this process.

Following segmentation, we used LN2_LAYERS from LayNii (v2.4.0; Huber et al., 2021a) to compute equi-volume cortical depths (Waehnert et al., 2014), cortical thickness, and cortical curvature for each gray matter voxel within the region of interest. LN2_LAYERS, LN2_MULTILATERATE, and LN2_PATCH_FLATTEN were then used to flatten the cortical patches (Huber et al., 2021a; Gulban et al., 2022). This process allows to flatten an originally folded 3D chunk of cortex to explore its cortical structure and functional organization at mesoscopic scale.

### 2.6 Functional data analysis: retinotopic mapping

Retinotopic data was processed using mrVista analysis software^2^ (Vista Lab, Stanford University): within run head motion was corrected and functional runs were aligned to the high-resolution T1-weighted structural image. Aligned functional data was then averaged and correlations analysis followed standard procedure (e.g., Zamboni et al., 2024; Welbourne et al., 2018). The resulting phase maps were visualized onto flat patches centered around the occipital pole and used to identify the following regions in both hemispheres: V1, V2, V3, and V4. The latter represents our region of interest (ROI) and focus of this study. Note that the flat patches generated in mrVista used the GM/WM segmentations from Freesurfer and are independent from the flattened cortical chunks obtained with LayNii (v2.4.0; Huber et al., 2021a).

Once V4 ROIs were identified, we used ANTs registration tool (Avants et al., 2009) to bring these back to T1w-EPI space by applying the transformation matrix described in the Structural Data Analysis section. The resulting V4 masks were then used to compute curvature-specific functional maps.

### 2.7 Functional data analysis: curvature mapping

The VASO sequence acquires images with Blood Oxygenation Level Dependent (BOLD) and Cerebral Blood Volume (CBV) contrast simultaneously in an interleaved fashion. We note that many of the analysis concerns highlighted for Ultra High-Field fMRI analysis (Polimeni et al., 2018) are applicable here. To perform appropriate preprocessing, first the nulled (VASO) and the not-nulled (BOLD) time-series were separated, for each run, each participant; and the steps described below were implemented to each time-series independently. Non-steady state images (first four time points – hence the long initial fixation period) were replaced by steady-state images (note that dummy scans are not acquired using this sequence). Motion correction was performed using SPM12 (v6906)^3^ and a 4th order spline was used to minimize blurring during resampling. Resulting motion parameters between the two contrasts were very similar, as expected, and below the nominal voxel resolution (1.2 mm isotropic). We then temporally up-sampled the nulled and not-nulled time-series with a factor of 2 (3dUpsample in AFNI using a 7th order polynomial interpolation) to restore the original time-series length. We then duplicated the first nulled timepoint to temporally match the nulled and not-nulled time-series. Dynamic division of nulled and BOLD volumes was performed using LN_BOCO (LayNii v2.4.0; Huber et al., 2021a) to generate VASO images with reduced BOLD contamination. Multiple runs were then averaged to minimize noise and computing demands. Temporal signal to noise ratio (tSNR) maps were also computed for quality control using AFNI (Cox and Hyde, 1997). General linear modeling, in terms of statistical parametric mapping, was computed using a 3-component design matrix consisting of a DC offset, a ramp, and a box-car stimulus profile reflecting the experimental design. No convolution with a haemodynamic response function was performed to the design matrix as this would assume that CBV and BOLD share the same profiles. Indeed, preclinical data has shown a significant delay in CBV response with respect to BOLD response following both onset and offset of somatosensory stimulation (Mandeville et al., 1999; Kennerley et al., 2005). While this delayed compliance, driven by slow relaxation of the venous walls following expansion/contraction, seems less obvious in human VASO studies (Hua et al., 2011; Lu et al., 2004; Huber et al., 2021b), caution should be taken when modeling the temporal dynamics of mechanisms underlying complex neurovascular coupling mechanisms. Statistical t-maps were then computed by contrasting stimulus (RF0 and RF4) versus baseline. Furthermore, to assess voxel-wise curvature preference, t-score maps were obtained by computing the difference in peak block response between visual stimuli (RF0 > RF4), with positive values indicating preference for curvature. An un-thresholded preference map for BOLD and VASO, respectively, was obtained by therefore assigning to each voxel within V4 the corresponding *t*-value, and its sign determined the stimulus preference (positive for curvature, negative for straight contours). Visualization of these maps onto flat patches allows to investigate the functional organization of curvature responses in human area V4. To further quantitatively compare the spatial organization of curvature maps between BOLD and VASO contrasts, we computed the principal component for each curvature map and collapsed voxel responses along the lead axis. A modular organization of curvature responses should result in a sinusoidal function (signal changes between curvature and straight contours responses) and deviations from this can be taken as evidence of reduced spatial specificity. Here, we note that our simple PCA analysis assumes linear banding in the surface distribution of the curvature preference values. This means that blurring is expected if this organization is more complex in shape. Despite this possible limitation, this approach represents a preliminary strategy to quantitatively assess spatial organization of curvature maps.

To provide a quantitative comparison of the signal properties between BOLD and VASO contrast, we followed Pizzuti et al. (2023) and computed an index of sensitivity and an index of specificity for each voxel within V4, for BOLD and VASO separately. Here, sensitivity reflects how strongly a voxel responds to any visual stimulus (regardless of the curvature property); specificity, on the other hand, corresponds to how well a voxel is tuned to curvature (RF0 > RF4).

For each contrast (BOLD, VASO) separately, sensitivity is computed as the Euclidean distance between the voxel response to RF0 and that to RF4. Therefore, given a vector with a *t*-value entry for each stimulus type, for each voxel within area V4, sensitivity is given by:


$$S\,e\,n\,s\,i\,t\,i\,v\,i\,t\,y=∥\vec{v}∥$$

Specificity was computed as:


$$S\,p\,e\,c\,i\,f\,i\,c\,i\,t\,y=1-\frac{\cos^{-1}\left(\hat{v}\cdot \hat{w}\right)}{\theta_{m\,a\,x}}$$

Where $\hat{v}$ is the Euclidean distance computed during the sensitivity step, $\hat{w}$is a reference winning vector (for example [0 1]) and θ is a scaling factor (i.e., the maximum angle determined by the dimensions available; so 45°). Specificity is then the additive inverse of the computed scaled angle. Given the properties of BOLD contrast, we expect this to show high sensitivity compared to VASO. However, its weighting toward large vasculature is expected to impact its specificity in resolving spatially refined functional responses within V4. Specificity values, how tuned a voxel response is to a specific stimulus feature (here curvature), are expected to span a narrower range compared to VASO.

## 3 Results

### 3.1 Functional data quality

**FIGURE 2:**
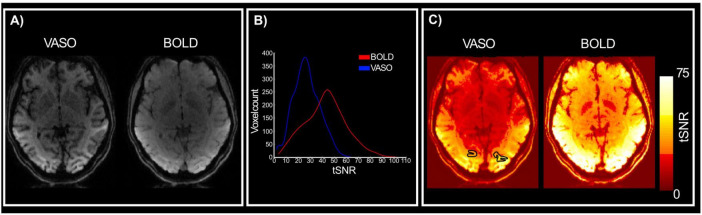
Quality assessment. **(A)** Mean VASO and BOLD images for a representative participant. Clear anatomical contrasts can be identified from the VASO images. **(B)** Distribution of VASO and BOLD tSNR values for the region of interest (V4) across participants. **(C)** VASO and BOLD tSNR maps for a representative participant.

To assess the quality of data using the optimized 3D EPI VASO sequence for use at 3T, we can first look at the image intensity distribution and contrast between gray matter and white matter boundaries. Figure 2 shows data from a representative participant (see Supplementary Figures 1–3 for individual participants); mean VASO and BOLD images are sharp and show minimal artifacts, with the VASO mean images enabling clear identification of anatomical structures (improved T1 contrast due to signal inversion). When looking at the distribution of tSNR within the region of interest, across participants, (Figures 2B, C) we notice that values are well above 25 for VASO and above 40 for BOLD. While lower tSNR for VASO is expected due to the signal inversion for blood nulling, the values reported here are comparable with others in the literature (e.g., Huber et al., 2023; Dresbach et al., 2024) and highlight the sufficient quality of functional data and feasibility of high resolution VASO acquisition at a clinically relevant field strength (3 Tesla).

**FIGURE 3:**
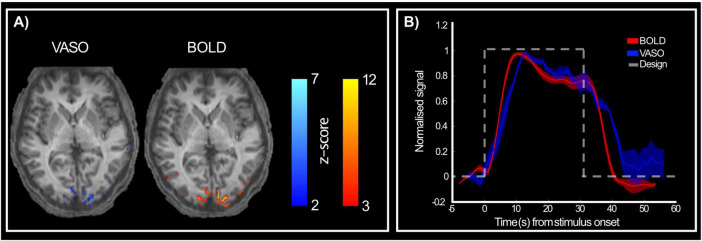
Activation maps following stimulus presentation. **(A)** VASO (right) and BOLD (left) show active voxels during stimulus presentation compared to rest period for a representative participant. BOLD contrast shows stronger responses than VASO, as expected. **(B)** Group-level (*n* = 5) normalized BOLD (red) and VASO (blue) signal in response to both stimuli (RF0 and RF4). Dashed gray line shows stimulus presentation design to highlight the difference in haemodynamic between the two imaging contrasts, with VASO showing a slightly delayed peak and delayed return to baseline compared to BOLD.

When looking at activation maps following stimulus presentation (Figure 3), BOLD contrast shows more activated voxels than VASO. However, counter intuitively, this likely reflects lower spatial specificity of BOLD and its greater weighting toward surface draining veins (Uğurbil et al., 2003; UIudag et al., 2009; Yacoub et al., 2005). Importantly, when comparing activation between VASO and BOLD, we can appreciate that this is relatively reduced for the VASO contrast, while BOLD maps show a wider spread of activated voxels beyond the gray matter boundary and expanding into cerebrospinal fluid (CSF) regions. These results suggest that 3T high spatial resolution VASO imaging methods provide reliable signal to investigate brain function, with the expected stronger signal changes for BOLD over VASO due to unwanted sensitivity to large draining veins of BOLD contrast.

Figure 3B shows the normalized average response time series (across stimuli and participants) for both BOLD and VASO measures. Both responses peak approximately 6 s after stimulus onset. After the initial peak, the responses plateau until stimulus offset (indicative of nonlinear neural–haemodynamic coupling; Martindale et al., 2005). We do observe a delayed return to baseline for the VASO response as evidence of expected delayed compliance (Kong et al., 2004).

### 3.2 Curvature response mapping in human V4

**FIGURE 4:**
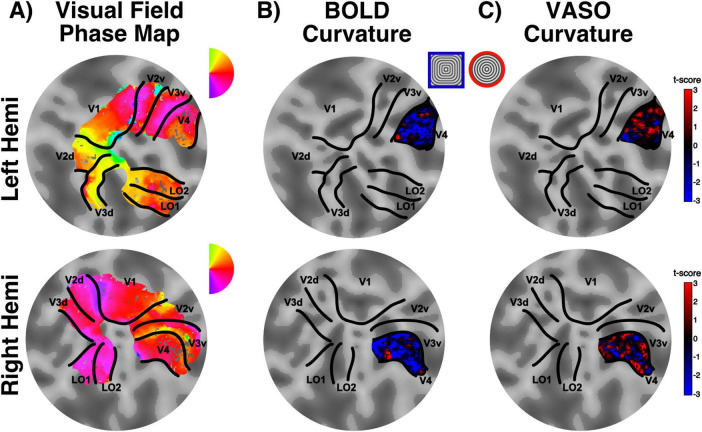
Curvature preference maps. **(A)** Retinotopically defined ROIs: flat patch obtained from the occipital pole overlaid with a color map detailing polar angle for left (top) and right (bottom) hemispheres. **(B)** Example of V4 curvature preference map for BOLD contrast in one representative participant. Red regions here show preference for curvature, whereas blue regions show preference for straight contours (stimulus legend inserted between maps). **(C)** Data as presented in **(B)**, here showing results for VASO contrast. Note the lower specificity of BOLD compared to VASO when identifying curvature-preference responses (higher proportion of voxels clustered around the zero t-score value).

We identified visual areas using standard retinotopic mapping procedures (Figure 4A) and determined area V4 as the region of interest for the curvature response mapping. Here, we measured the response to curved stimuli (RF0 – concentric circles) compared to straight contours stimuli (RF4 – concentric squares) and looked at the spatial organization of this voxel-wise preferential response in BOLD and VASO contrasts, respectively (Figures 4B, C). Statistical maps for curvature responses appear to show a stripe-like pattern across the cortical surface for both BOLD and VASO contrasts within area V4, in both hemispheres. While there is variability across the participants (see Supplementary Figures 1–3 for individual maps), the overall spatial modularity of curvature responses is consistent. Interestingly, these “patches” of curvature-preference seem qualitatively better defined in the VASO contrast, compared to BOLD. This would support the idea that, while BOLD offers higher sensitivity (higher signal overall), it lacks in specificity (here, differentiating between the two visual features, curved and straight contours).

**FIGURE 5:**
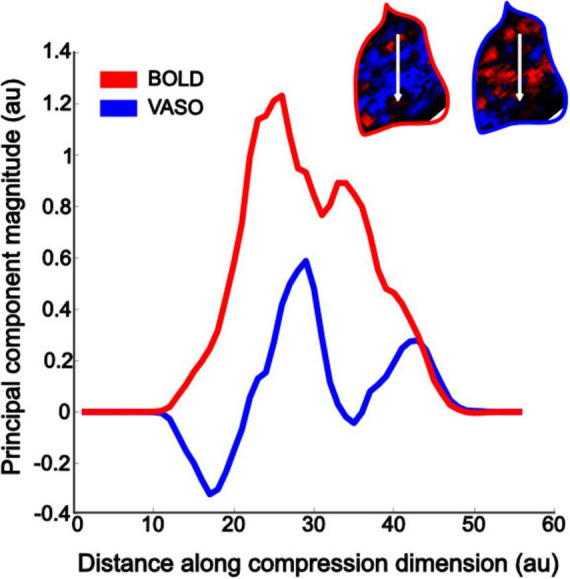
Curvature preference maps specificity quantification. Collapsing the curvature preference data across the principal component (white arrow in the top right inserts, for BOLD – red contour map, and VASO – blue contour map, respectively) allows to quantify the difference in modular organization between the two imaging contrasts. The VASO contrast shows a clear sinusoidal modulation (blue line), which is instead strongly dominated by a main peak in the BOLD data (red line).

To quantify this apparent difference in curvature map specificity between BOLD and VASO, we computed the curvature preference maps principal component and collapsed the map’s values along the primary axis. A modular organization of curvature responses should result in a sinusoidal component, highlighting the changes in response between preference for curvature and preference for straight contours. Deviations from this sinusoidal function would represent a lack of specificity in signal response to the two conditions tested. Indeed, when comparing the principal component for VASO and BOLD (Figure 5), this is what we observe: VASO provides a cleaner definition of the changes in curvature preference across voxels in area V4 than BOLD contrast.

**FIGURE 6:**
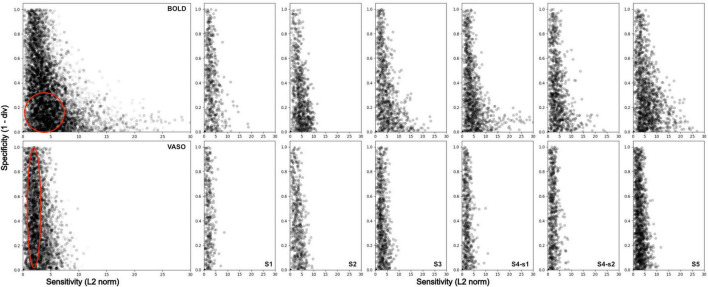
Sensitivity and Specificity scatterplot for BOLD (top row) and VASO (bottom row) in area V4 for all participants (first column), and for each subject separately (columns 2–7). BOLD data show a wider range of values along the sensitivity axis and a higher density of values located around the bottom left corner of the scatterplot (low specificity).

To further quantify the differences between the two imaging contrasts, we computed two indices for each voxel within V4: sensitivity, how strongly a voxel responds to any stimulus type; and specificity, how well a voxel responds to a curved contour stimulus. Figure 6 shows a scatter plot of the sensitivity-specificity values for BOLD (top row) and VASO (bottom row) for all participants (column 1, columns 2–7 for individual participants). For BOLD contrast, the distribution shows a wide range of sensitivity values (long tail along the x-axis) and a narrow band for the specificity values (compressed distribution along the y-axis), in line with previous observations (Pizzuti et al., 2023). For VASO contrast, we see a much-reduced spread of sensitivity values, in agreement with recent findings (Huber et al., 2017; Beckett et al., 2020) suggesting VASO suffers from reduced signal sensitivity when compared to BOLD contrast. However, distribution of specificity values outranges that observed in BOLD, supporting the idea that VASO contrast provides greater interpretability of the fMRI signal.

Our surface maps, alongside PCA analysis, provide evidence for the existence of curvature domains in human V4, and are consistent with the preclinical measures of systematic maps of curvature representation in macaques V4 (Tang et al., 2020; Hu et al., 2020; Yue et al., 2014). We take this finding one step further by exploring whether curvature domains span the cortical depth and follow a modular functional organization structure.

**FIGURE 7:**
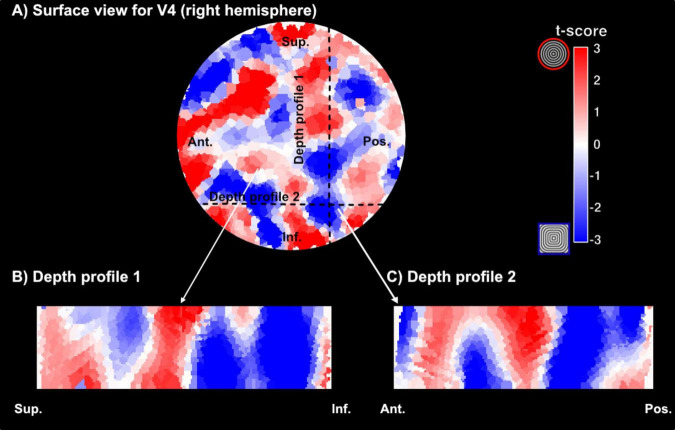
Examples of curvature domains across the cortical depth. **(A)** Surface visualization of a section of area V4 (right hemisphere) and corresponding curvature preference maps (red – preference for curved contours, blue – preference for straight contours). The section here has been upsampled to a 0.2 mm isotropic resolution. **(B,C)** Visualization of curvature preference maps for two different cross-sections of cortex for VASO contrast in one representative participant.

Here, we used the flattening algorithm (Gulban et al., 2022) and 3D rendering visualization tools (Sullivan and Kaszynski, 2019) to sample curvature responses across the cortical depth. Figure 7 shows our columnar results for one representative participant for VASO contrast. Qualitatively, we observe small patches in area V4, with preference for curvature and straight contours alternating throughout the cortical depth. The spatial organization can be fully observed in the rotating animation accompanying Figure 7 available at this link^4^. Furthermore, when comparing this functional organization between the two contrasts, and in line with the measures of sensitivity and specificity reported above, we observe that VASO results are more specific than BOLD. While the overall spatial organization of curvature-selective features will require further investigation to be fully established (with more averaging), we report first, exciting data suggesting modular organization of curvature domains in human V4 can be measured at clinically relevant MR field strengths.

## 4 Discussion

This study shows the feasibility of using Vascular Space Occupancy (VASO) fMRI at 3 Tesla to investigate the mesoscopic functional organization of visual area V4 in humans. We provide evidence of curvature domains in human V4 that is consistent with findings from non-human primates (Tang et al., 2020; Hu et al., 2020). While previous functional MRI studies (both at 3 and 7T) in macaques and humans have reported patches of visual cortex – zones much larger than the mesoscopic domains – are involved in processing curvature information (Yue et al., 2014; Yue et al., 2020), our work advances the understanding of the role of mid-level area V4 in processing curvature and shape features. Indeed, the role of this visual area, situated in the ventral visual pathway, is still elusive. This is partly due to the richness of response properties shown by V4 neurons, such as preferences for color (Zeki, 1973; Conway et al., 2007), luminance, texture (Arcizet et al., 2008; Arcizet et al., 2009; Merigan, 2000), as well as orientation (Ghose and Ts’o, 1997), curvature (Pasupathy and Connor, 1999, 2001), motion (Mountcastle et al., 1987), and binocular disparity (Watanabe et al., 2002). The large receptive fields of V4 neurons allow integration of information across spatially separated stimuli (Cox et al., 2013). This capability is essential for tasks such as surface completion and object recognition, where the brain must synthesize information from various parts of a visual scene. As noted by Roe et al. (2012), V4 serves as a mid-tier area in the ventral visual pathway, bridging the gap between early visual areas like V1 and higher-order areas involved in object recognition. This hierarchical arrangement allows for a progressive refinement of visual information, with V4 playing a crucial role in transforming basic visual inputs into more complex representations. A columnar architecture supports this transformation by ensuring that neurons with similar tuning properties are grouped together, facilitating efficient information processing. Tootell and Nasr (2017) demonstrated that this segregation is evident using high spatial resolution BOLD fMRI at ultra-high field strength (7T). They investigated whether a columnar architecture aids segregation of magnocellular (M) and parvocellular (P) pathways in extrastriate visual areas (V2-V4). It is known that these two streams independently extend from the retina through early visual cortex, each showing distinct functional properties (Hubel and Livingstone, 1987; Zeki and Shipp, 1988; Felleman and Van Essen, 1991). Tootell and Nasr (2017) elegantly show that area V4 presents a radial (i.e., columnar) organization for visual features such as color and binocular disparity, as well as luminance contrast. This characteristic architecture seems to enable area V4 to handle inputs from various sources and integrate them into coherent representations. In a similar fashion, Ai et al. (2024) used high spatial resolutions BOLD fMRI at 7T to explore the columnar organization of response properties to texture in human visual cortex. Interestingly, while V4 modularity seems to emerge primarily for color processing, the connectivity patterns of V4 neurons reveal the significance of columnar organization in establishing functional networks within the visual cortex, with feedback connection between area V4 and V2 supporting processing of texture information.

While these studies start to uncover modular organization of area V4 in humans, the exploration of columnar structure of curvature processing in V4 has been limited to non-human primates. Research by Tang et al. (2020) & Hu et al. (2020) identified specific domains within macaques V4 that are tuned for curvature, where this specialization allows V4 to contribute significantly to the perception of object boundaries and contours, critical for object recognition and categorisation. Our work represents the first attempt at mapping curvature responses in human V4 using VASO fMRI at clinically relevant field strengths (3T). Based on a qualitative assessment of our depth-dependent analysis for curvature preference, our results are consistent with those in non-human primates, but further work on the spatial scale of the domains and their functional specificity will be required to confirm that the pattern of organization is homologous.

One of the primary advantages of VASO fMRI, over more commonly used BOLD fMRI, is its sensitivity to changes in CBV, which more closely reflect neurovascular coupling (Huber et al., 2021b). Recent studies (Huber et al., 2023) have demonstrated that VASO can effectively capture laminar activity in cortical regions, offering insights into the functional organization of the brain at a finer spatial resolution than traditional BOLD imaging. Furthermore, Huber et al. (2023) emphasized the potential for VASO fMRI at 3T to achieve spatial resolution comparable to that of higher field strengths (7T and 9.4T). Supporting this principle, we show that our distributions of sensitivity and specificity values align with metrics reported by Pizzuti et al. (2023) investigating the columnar organization of axis of motion in human MT+ at 7T. Specifically, we show that BOLD contrast is characterized by larger sensitivity values, confounded by a weighting toward large pial vasculature (Uğurbil et al., 2003; Yacoub et al., 2005; Uludağ et al., 2009), and also a large proportion of voxels with low specificity values. VASO, on the other hand, shows lower sensitivity, consistent with this contrast expected to be mostly driven by microvasculature and less affected by large pial vasculature. However, it is important to stress that the BOLD contamination correction applied in this work [a dynamic division between the BOLD and VASO signals (Huber et al., 2014)], may still result in residual BOLD contamination. The transverse venous sinus, a large blood vessel, is frequently found in close proximity to area V4 in humans, thus greatly impacting fMRI measurements in this region (Winawer et al., 2010). A recent study by Chen et al. (2024) proposed the use of an ICA-based method to mitigate contaminations in functional MR signals from large draining veins. This method leverages the characteristic temporal delays of BOLD signals across cortical depths (with characteristic delayed peaks in superficial layers) to differentiate between BOLD and non-BOLD signals. Application of this technique should be considered in the future to further address possible residual BOLD contaminations in VASO.

Importantly, VASO shows enhanced specificity compared to BOLD, which is a critical aspect when investigating response profiles to visual properties at high spatial resolution. This is also evidenced from our PCA results highlighting the defined profile observed in curvature preference maps obtained using VASO contrast, compared to BOLD. The latter shows a less pronounced sinusoidal profile, with one prominent peak, suggesting a lower proportion of responses differentiating between the two stimulus types used in this study, in line with the observed lower specificity values. These findings therefore are not only important from a neuroscientific perspective, but also highlight the feasibility of using high-resolution VASO acquisition at clinically relevant field strengths scanners. Indeed, 3T scanners are widely available in both clinical and research settings, and implementation of VASO will widen accessibility to explore complex brain functions with high spatial specificity, especially when using paradigms or clinical populations that would be otherwise challenging at higher field strengths (7T+).

Despite these advantages challenges remain in the application of high-resolution VASO fMRI at 3T. One notable issue is the inherent low signal-to-noise ratio that can hinder the ability to detect subtle changes in CBV associated with neural activity. While our data, consistent with previous research at ultra-high field strength (Dresbach et al., 2024), shows overall lower tSNR values for VASO contrast compared to BOLD, it is worth notice that this imbalance is expectedly due to thermal noise in contrast to physiological noise as it is at higher field strengths (Wald and Polimeni, 2017). Furthermore, when compared to tSNR values at 7T, VASO at lower field strengths shows an advantage. This can be linked to the known longer T2* and stronger T1 contrast at 3T, which can mitigate limitations inherent of higher field strengths such as reduced magnetization in inversion-recovery sequences like VASO (Huber et al., 2023). One promising approach to enhance SNR is the application of NOise Reduction with DIstribution Corrected (NORDIC) PCA (Vizioli et al., 2021). NORDIC operates by leveraging the low-rank structure of fMRI data, which allows for the effective suppression of Gaussian noise without introducing excessive spatial smoothing. Knudsen et al. (2023) reported that application of NORDIC denoising to high-resolution BOLD fMRI data acquired at 3T resulted in an average increase in tSNR by a factor of approximately three in their sample. Similarly, evaluation of this processing technique at 7T has shown reduced thermal noise contribution and overall enhanced quality of fMRI data (Faes et al., 2024; Dowdle et al., 2023).

It is important to note that the use of 1.2 mm isotropic voxels here is relatively coarse for cortical-depth analyzes and most layer-fMRI studies use voxel sizes of around 1–0.8 mm isotropic. Moving to higher spatial resolution acquisition will inherently impact SNR, potentially affecting the reliability of the acquired data. Indeed, smaller voxels are more susceptible to physiological noise, which can obscure the fMRI signal (Liu et al., 2023; Markuerkiaga et al., 2020). Furthermore, higher spatial resolution typically requires longer scan times, either due to longer repetition time or a need for a greater number of trials to ensure adequate SNR (Blazejewska et al., 2019). Advanced hardware and sequence optimisation could help address this trade-off between spatial resolution, SNR, and scan time (e.g., Huber et al., 2023; Polimeni et al., 2018), combined with post-processing strategies such as NORDIC denoising (Vizioli et al., 2021). It is noted here that our sample size is of *N* = 5, relatively small albeit within the range used in previous fMRI studies at higher field strength (e.g., Yacoub et al., 2008; De Martino et al., 2013; Pizzuti et al., 2023; Haenelt et al., 2023b); and the total number of trials examined were 280. To increase SNR one could consider increasing sample size and trial numbers. Observing the haemodynamic response obtained by the long stimulus from both BOLD and VASO contrasts (Figure 3B), it is easy to identify an initial peak in response, followed by a plateau in response, and return to baseline following cessation of stimulation. This is in line with data showed by Martindale et al. (2005) demonstrating, in preclinical models, that a traditional linear convolutional model is not adequate in describing long stimuli. Pfeuffer et al. (2003) suggest that the early onset component of the haemodynamic response is linked to gray matter (and arterial) origin, while the plateau is linked to veins. Interestingly, Rosengarten et al. (2003) further suggest that the two compartments might relate to feedforward (peak response) and feedback (plateau response) mechanisms. While understanding the neurophysiology underlying these responses is beyond the scope of the current study, it is a question that remains important and needs addressing as using accurate modeling of haemodynamic response is crucial for understanding brain function and connectivity. Thus, a viable step to further investigate the modular architecture of area V4 in humans could be to use shorter trials (i.e., capturing only the peak-response) and further combine data acquisition with NORDIC denoising.

We also note that this investigation of functionally specialized domains is preliminary and that we capture only functional preferences to curvature compared to straighter components of the stimulus. We have not therefore examined chromatic properties of visual stimuli that have been shown to be processed in domains that differ from those processing orientation and curvature (Tang et al., 2020; Hu et al., 2020). Capturing V4 responses to all three stimulus properties in future work should allow the idea that V4 in macaque and human are homologous to be examined. The extent of visual field representation in a contiguous map in ventral visual cortex varies between macaque (one quarter field in ventral and dorsal V4, respectively; Zeki, 1971) and human (a contiguous hemifield representation in a ventral location; Winawer and Witthoft, 2015) could be one reason to doubt that functional properties of V4 in human and macaque may differ. In addition to exploring further stimulus dimensions, investigating the observed inter-individual variability of the modular organization of curvature preferences in V4 could elucidate whether these relate to individual differences in visual perception. While changes in functional and structural organization can be linked to neurodegenerative diseases, variability is also observed across healthy individuals. Further addressing the relationship between individual differences in mid-level areas architecture and how these relate to differences in visual perception can inform our knowledge of feature processing and object representation. This in turn can be used to inform novel computational models of visual processing and advance object representation algorithms.

## Data Availability

The raw data supporting the conclusions of this article will be made available by the authors, without undue reservation.

## Footnotes

1
https://surfer.nmr.mgh.harvard.edu/

2
https://github.com/vistalab/vistasoft/

3
https://www.fil.ion.ucl.ac.uk/spm/software/spm12

4 https://drive.google.com/drive/folders/1Gf2jonruijIw5ZNP1J1ORTrYh KJFqxo-.

## Supplementary material

Supplementary Figure 1 Summary data for participants 1 and 2. **(A)** Visual field maps renderings on flat patches for left (rows one and three), and right (rows two and four) hemispheres. Here the region of interest, visual area V4, can be identified ventrally. **(B)** V4 curvature preference map for BOLD contrast, with warm colors indicating preference for curvature (RF0 – concentric circles insert) and cool colors indicating preference for straight contours (RF4 – concentric squares insert). **(C)** V4 curvature preference map for VASO contrast – color map same as in panel **(B)**. A higher definition of structure in curvature mapping emerges here. **(D)** Principal component analysis output when collapsing the curvature maps **(B,C)** along the main axis of signal change. While these show variability across participants and hemispheres, VASO contrast (blue line) generally shows a sinusoidal modulation, supporting higher signal specificity (here the case for Subject 2, while Subject 1 shows better delination for BOLD in the left hemisphere, and comparable results for the right hemisphere). **(E)** tSNR map for BOLD (top) and VASO (bottom), thresholded as indicated in Figure 2 (0–75). Consistent with other findings, BOLD shows higher tSNR values compared to VASO. **(F)** Activation maps for BOLD (top) and VASO (bottom) during stimulus presentation. Note that here Subject 1 performed 4 runs, while Subject 2 only performed one functional run. Despite this imbalance, response to visual stimuli is defined in both participants’ visual cortex.

Supplementary Figure 2 Summary data for participants 3 and 5. **(A)** Visual field maps renderings on flat patches for left (rows one and three), and right (rows two and four) hemispheres. Here the region of interest, visual area V4, can be identified ventrally. **(B)** V4 curvature preference map for BOLD contrast, with warm colors indicating preference for curvature (RF0 – concentric circles insert) and cool colors indicating preference for straight contours (RF4 – concentric squares insert). **(C)** V4 curvature preference map for VASO contrast – color map same as in panel **(B)**. A higher definition of structure in curvature mapping emerges here. **(D)** Principal component analysis output when collapsing the curvature maps **(B,C)** along the main axis of signal change. While these show variability across participants and hemispheres, VASO contrast (blue line) generally shows a sinusoidal modulation, supporting higher signal specificity (Subject 5 shows similar profiles for BOLD and VASO in the left hemisphere). **(E)** tSNR map for BOLD (top) and VASO (bottom), thresholded as indicated in Figure 2 (0–75). Consistent with other findings, BOLD shows higher tSNR values compared to VASO. **(F)** Activation maps for BOLD (top) and VASO (bottom) during stimulus presentation.

Supplementary Figure 3 Summary data for participant 4, sessions 1 and 2. **(A)** Visual field maps renderings on flat patches for left (rows one and three), and right (rows two and four) hemispheres. Here the region of interest, visual area V4, can be identified ventrally. **(B)** V4 curvature preference map for BOLD contrast, with warm colors indicating preference for curvature (RF0 – concentric circles insert) and cool colors indicating preference for straight contours (RF4 – concentric squares insert). **(C)** V4 curvature preference map for VASO contrast – color map same as in panel **(B)**. A higher definition of structure in curvature mapping emerges here. **(D)** Principal component analysis output when collapsing the curvature maps **(B,C)** along the main axis of signal change. While these show variability across participants and hemispheres, VASO contrast (blue line) generally shows a sinusoidal modulation, supporting higher signal specificity (Subject 4 shows similar profiles for BOLD and VASO in the right hemisphere of session 1). **(E)** tSNR map for BOLD (top) and VASO (bottom), thresholded as indicated in Figure 2 (0–75). Consistent with other findings, BOLD shows higher tSNR values compared to VASO. **(F)** Activation maps for BOLD (top) and VASO (bottom) during stimulus presentation.
