# Supplementary figures and images for: Mapping curvature domains in human V4 using CBV-sensitive layer-fMRI at 3T
Source: Front Neurosci. 2025 Feb 26;19:1537026. doi: 10.3389/fnins.2025.1537026 (PMC11897262; doi:10.3389/fnins.2025.1537026)

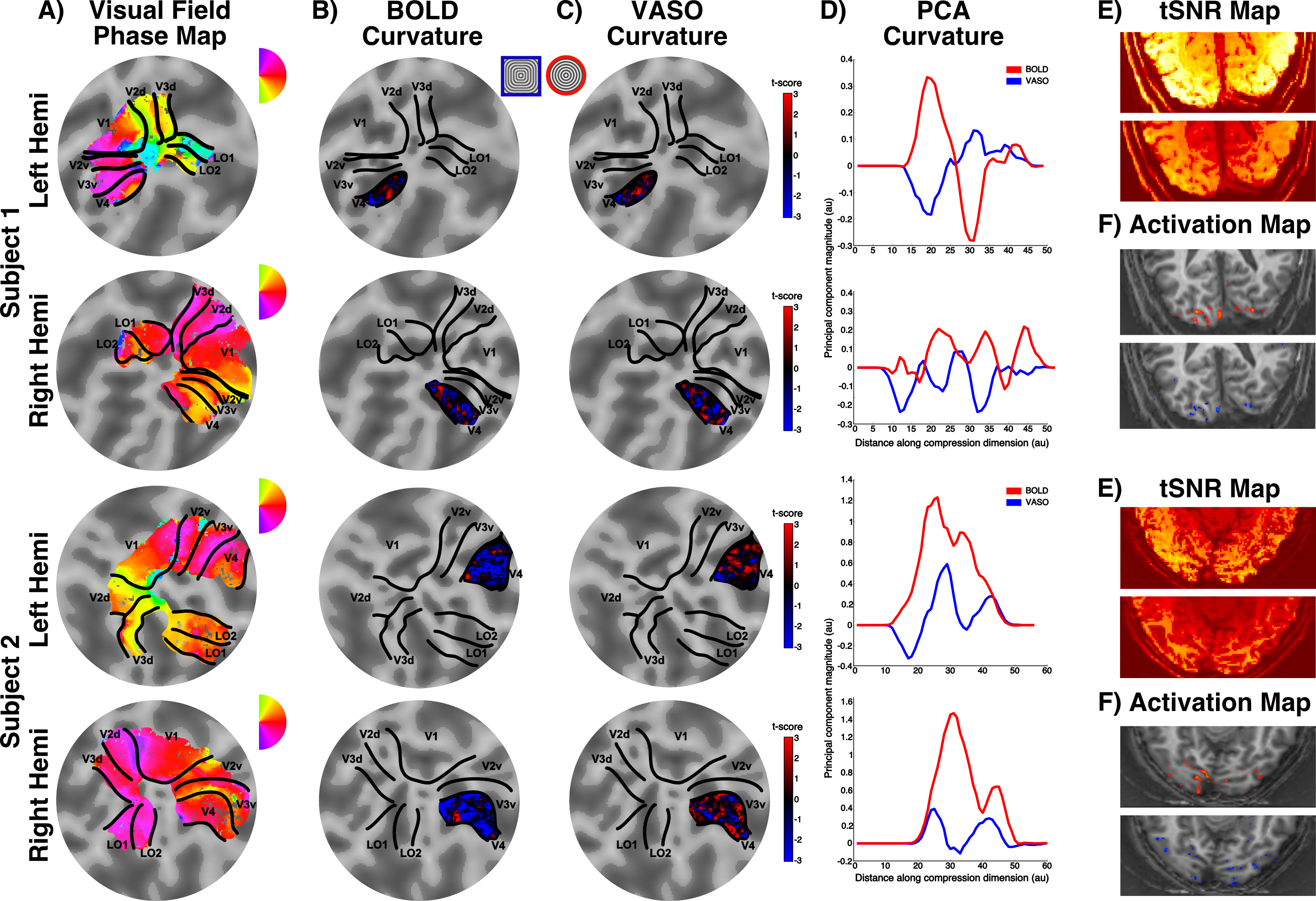

Supplement: Supplementary Figure 1 — Summary data for participants 1 and 2. (A) Visual field maps renderings on flat patches for left (rows one and three), and right (rows two and four) hemispheres. Here the region of interest, visual area V4, can be identified ventrally. (B) V4 curvature preference map for BOLD contrast, with warm colors indicating preference for curvature (RF0 – concentric circles insert) and cool colors indicating preference for straight contours (RF4 – concentric squares insert). (C) V4 curvature preference map for VASO contrast – color map same as in panel (B). A higher definition of structure in curvature mapping emerges here. (D) Principal component analysis output when collapsing the curvature maps (B,C) along the main axis of signal change. While these show variability across participants and hemispheres, VASO contrast (blue line) generally shows a sinusoidal modulation, supporting higher signal specificity (here the case for Subject 2, while Subject 1 shows better delination for BOLD in the left hemisphere, and comparable results for the right hemisphere). (E) tSNR map for BOLD (top) and VASO (bottom), thresholded as indicated in Figure 2 (0–75). Consistent with other findings, BOLD shows higher tSNR values compared to VASO. (F) Activation maps for BOLD (top) and VASO (bottom) during stimulus presentation. Note that here Subject 1 performed 4 runs, while Subject 2 only performed one functional run. Despite this imbalance, response to visual stimuli is defined in both participants’ visual cortex. [file Image_1.jpeg]

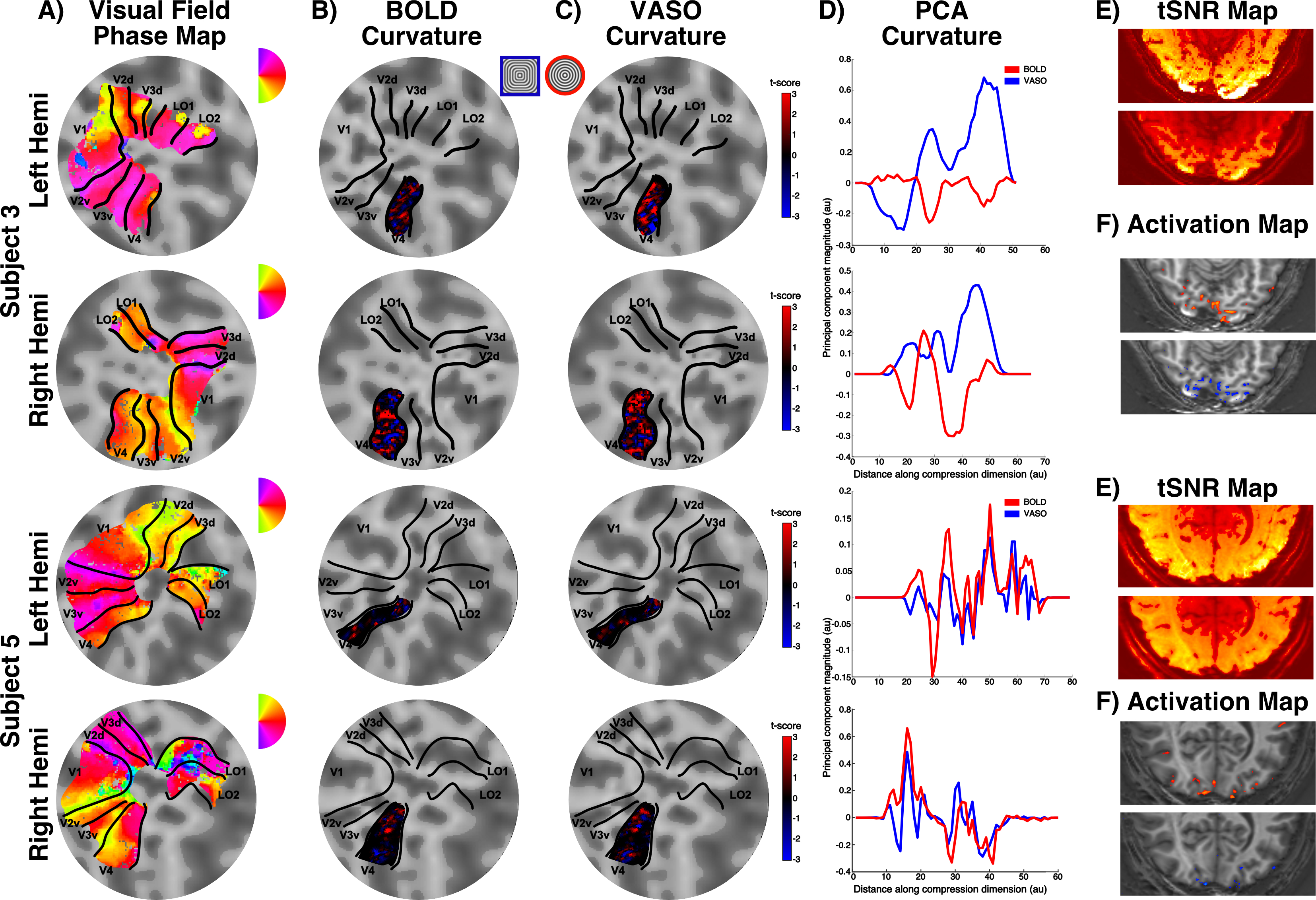

Supplement: Supplementary Figure 2 — Summary data for participants 3 and 5. (A) Visual field maps renderings on flat patches for left (rows one and three), and right (rows two and four) hemispheres. Here the region of interest, visual area V4, can be identified ventrally. (B) V4 curvature preference map for BOLD contrast, with warm colors indicating preference for curvature (RF0 – concentric circles insert) and cool colors indicating preference for straight contours (RF4 – concentric squares insert). (C) V4 curvature preference map for VASO contrast – color map same as in panel (B). A higher definition of structure in curvature mapping emerges here. (D) Principal component analysis output when collapsing the curvature maps (B,C) along the main axis of signal change. While these show variability across participants and hemispheres, VASO contrast (blue line) generally shows a sinusoidal modulation, supporting higher signal specificity (Subject 5 shows similar profiles for BOLD and VASO in the left hemisphere). (E) tSNR map for BOLD (top) and VASO (bottom), thresholded as indicated in Figure 2 (0–75). Consistent with other findings, BOLD shows higher tSNR values compared to VASO. (F) Activation maps for BOLD (top) and VASO (bottom) during stimulus presentation. [file Image_2.jpeg]

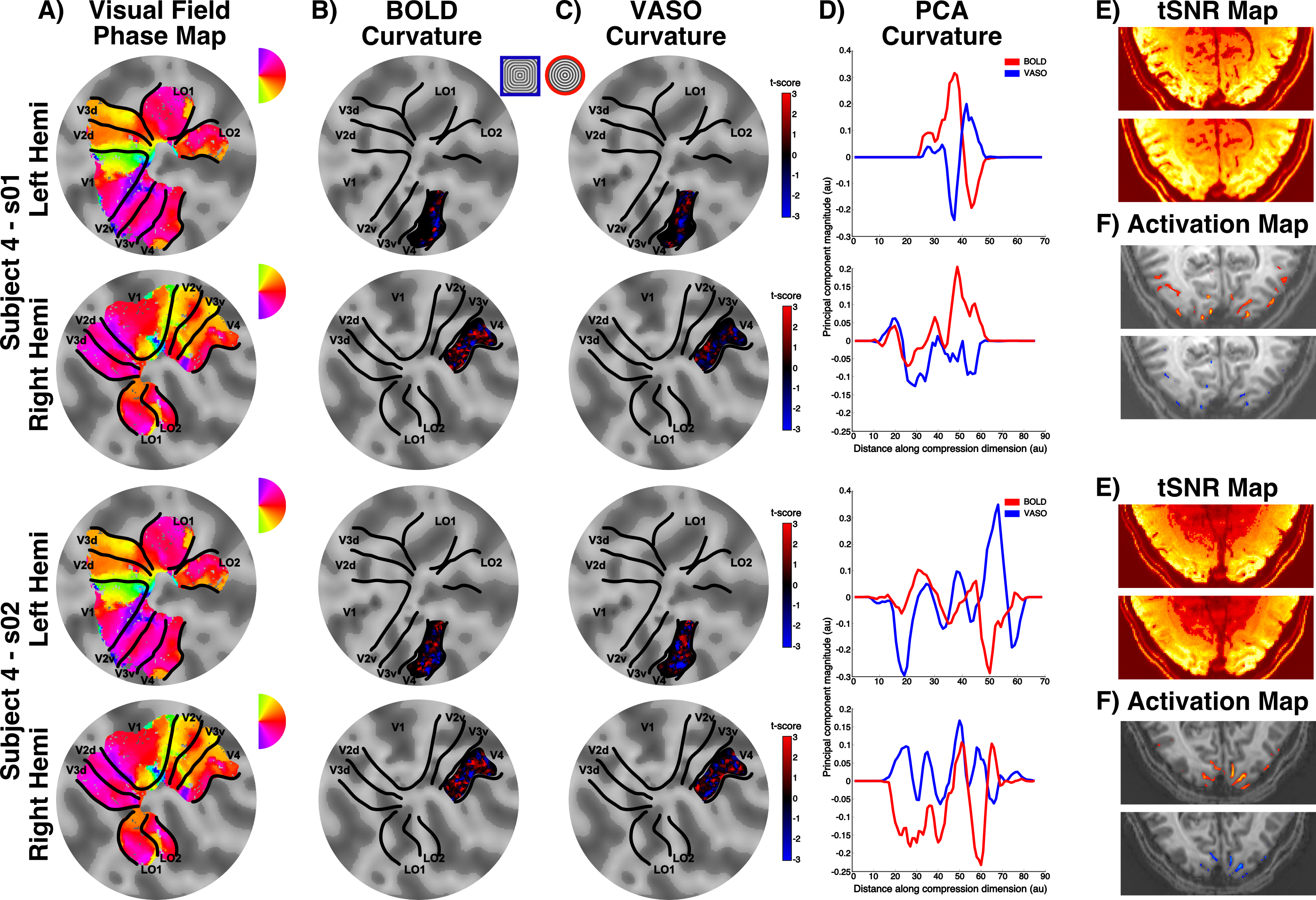

Supplement: Supplementary Figure 3 — Summary data for participant 4, sessions 1 and 2. (A) Visual field maps renderings on flat patches for left (rows one and three), and right (rows two and four) hemispheres. Here the region of interest, visual area V4, can be identified ventrally. (B) V4 curvature preference map for BOLD contrast, with warm colors indicating preference for curvature (RF0 – concentric circles insert) and cool colors indicating preference for straight contours (RF4 – concentric squares insert). (C) V4 curvature preference map for VASO contrast – color map same as in panel (B). A higher definition of structure in curvature mapping emerges here. (D) Principal component analysis output when collapsing the curvature maps (B,C) along the main axis of signal change. While these show variability across participants and hemispheres, VASO contrast (blue line) generally shows a sinusoidal modulation, supporting higher signal specificity (Subject 4 shows similar profiles for BOLD and VASO in the right hemisphere of session 1). (E) tSNR map for BOLD (top) and VASO (bottom), thresholded as indicated in Figure 2 (0–75). Consistent with other findings, BOLD shows higher tSNR values compared to VASO. (F) Activation maps for BOLD (top) and VASO (bottom) during stimulus presentation. [file Image_3.jpeg]
